# The Effect of Multiprobiotics on Memory and Attention in Fibromyalgia: A Pilot Randomized Controlled Trial

**DOI:** 10.3390/ijerph18073543

**Published:** 2021-03-29

**Authors:** Diana Cardona, Pablo Roman, Fernando Cañadas, Nuria Sánchez-Labraca

**Affiliations:** 1 Department of Nursing, Physiotherapy and Medicine, Health Research Center (CEINSA), University of Almeria, 04120 Almeria, Spain; dcardona@ual.es (D.C.); msl397@ual.es (N.S.-L.); 2 Department of Psychology, University of Almeria, 04120 Almeria, Spain; jcanadas@ual.es

**Keywords:** probiotics, memory, attention, microbiota, gut–brain axis, gastrointestinal microbiome, fibromyalgia

## Abstract

Fibromyalgia syndrome (FMS) is a chronic, generalized and diffuse pain disorder accompanied by cognitive deficits such as forgetfulness, concentration difficulties, loss of vocabulary and mental slowness, among others. In recent years, FMS has been associated with altered intestinal microbiota, suggesting that modulating gut microbiota (for example, through probiotics) could be an effective therapeutic treatment. Thus, the aim of the present study was to continue exploring the role of probiotics in cognitive processes in patients with FMS. A pilot randomized controlled trial was conducted in 31 patients diagnosed with FMS to compare the effects of a multispecies probiotic versus a placebo on cognitive variables (memory and attention) after eight weeks. Results showed that treatment with a multispecies probiotic produced an improvement in attention by reducing errors on an attention task, but it had no effect on memory. More specifically, a tendency to reduce errors of omission (Go trials) during the Go/No-Go Task was observed after treatment. These findings, along with our previous results in impulsivity, underline the relevance of using probiotics as a therapeutic option in FMS, although more research with a larger sample size is required.

## 1. Introduction

Fibromyalgia syndrome (FMS) is a chronic, generalized and diffuse pain disorder accompanied by symptoms such as morning stiffness, fatigue, depression and sleeping disorders [1]. Another prevalent complaint is cognitive deficits such as forgetfulness, concentration difficulties, loss of vocabulary and mental slowness, among others [2,3]. Some previous research found that FMS patients show poor performance in some executive functions [4], such as concentration, working memory deficits [5] and reduced ability to inhibit irrelevant information [6], as well as low cognitive flexibility and poor decision-making [4]. Likewise, in these patients, there is also less brain activation in the cortical structures of the inhibition network (specifically in the areas involved in response selection/motor preparation) and the attention network [7].

Recently, FMS has been associated with altered intestinal microbiota [8], as well as with chronic widespread musculoskeletal pain, a symptom of FMS which has shown reduced diversity in the microbiome, particularly of *Coproccocus*, indicating the involvement of the gut microbiota [9]. The gut microbiota plays an important role in different physiological functions, exerting effects from energy metabolism to psychiatric well-being [10]. Research has documented lower levels of *Bifidobacterium* and higher levels of *Enterococcus* spp. in these patients [11]. Furthermore, it has been stated that the higher the aerobic enterococcal count, the worse the neurological and cognitive deficits, such as nervousness, memory loss, forgetfulness and confusion [12]. This is related to the gut–brain axis pathway, which is a bidirectional communication network between the brain and the gut microbiota that occurs via three different pathways: neural, endocrine and immune [13]. It is worth mentioning that neural communication takes place through the vagus nerve and the enteric nervous system (ENS), while endocrine communication occurs via the production of hormones such as cortisol, and immune system communication takes place via the modulation of cytokines [14,15]. In this context, bacterial products activate the ENS [16] and stimulate primary afferent nerves, as well as bacterial metabolites that cause behavioral changes [17]. For these reasons, the gut–brain axis, which allows gut bacteria to affect the central nervous system (for example, with probiotic administration), has been used as a treatment option for a variety of health and mental disorders [18].

Probiotics are defined as live microorganisms which, when administered in adequate amounts, confer a health benefit on the host [19]. Probiotics have been shown to specifically catalyze oligosaccharides, increasing short-chain fatty acid (SCFA) production [20]. SCFAs are metabolic byproducts of the anaerobic fermentation of dietary carbohydrates and some amino acids, and they play a variety of roles in health maintenance, not only in the intestine as an energy source that improves transit, but also in the immune system [21]. Fibromyalgia (FM) patients have an altered composition of SCFAs, and *Parabacteroides merdae* increases neurotransmitters in FMS patients, which could explain the cognitive dysfunction [22].

In fact, FMS and irritable bowel syndrome (IBS) are common co-occurring disorders [23] for which modulation of the gut microbiota is a treatment strategy [24]. Moreover, FMS is frequently associated with other immuno-rheumatic diseases, such as chronic fatigue syndrome [25], which appears to improve after probiotics administration [26], or rheumatoid arthritis, in which probiotics also improve symptoms [27]. However, even though the gut microbiota may play a role in FMS, according to a recent systematic review, the data are insufficient [28], and more research is required to obtain conclusive answers in relation to the effectiveness of dietary interventions [29].

According to all of the above, changes in gut microbiota could be involved in FMS, so modulating the gut microbiota is a therapeutic treatment that needs to be explored. Therefore, we carried out a pilot study on the effect of multispecies probiotics on the cognitive and emotional symptoms of FMS [30]. In the first part of this study, we showed the beneficial effects of probiotics on impulsivity [31]. In this context, the current study aims to continue exploring the role of probiotics in cognitive processes in patients with FMS, specifically the effects of a multispecies probiotic on attention and memory function in FMS patients. Given the role of gut microbiota in central nervous system functions, we expect that oral intake of probiotics will have beneficial effects on memory and attention in FM.

## 2. Materials and Methods

### 2.1. Study Design and Participants

This study is part of a large, double-blinded study and a parallel group design that was registered with ClinicalTrial.gov (NCT02642289) and approved by the Human Research Ethics Committee of the University of Almeria (Spain). The study protocol and recruitment procedure have been previously described [30]. Fibromyalgia patients were recruited from the Almeria Fibromyalgia Association (AFIAL—Spain) or from El Ejido Fibromyalgia Association (AFIEL—Spain) and were diagnosed at least 1 year before entering the study according to the criteria of the American College of Rheumatology [1,32]. Exclusion criteria involved: (1) use of antibiotics and nutritional supplements, (2) allergies, (3) current participation in other psychological or medical studies, (4) being pregnant or breastfeeding, (5) severe intestinal disease and (6) meeting the criteria for psychiatric disorders other than depression and/or anxiety. More information about the participants’ characteristics can be found in the first part of the study [31].

### 2.2. Procedure 

Participants were randomly assigned to the following groups: experimental (ERGYPHILUS Plus (Laboratorios NUTERGIA S.L., San Sebastián, Spain)), which contained *Lactobacillus rhamnosus GG*, *L. paracasei*, *L. acidophilus* and *Bifidobacterium bifidus* (revivification of 6 million germs per capsule, 4 capsules per day, *n* = 16), or placebo (*n* = 15). The placebo capsules were composed of cellulose and provided by Complementos Fitonutricionales S.L. (Spain). The evaluation was performed both before the treatment (baseline) and after 8 weeks of treatment (post-intervention). More information about the procedure can be found in the first part of the study [31]. The duration of treatment was selected according to similar, previous research [33,34]. 

The selected probiotic species have been used previously to improve functions related to the gut–brain axis [33,35,36] and are therefore expected to be capable of attenuating the cognitive and emotional changes caused by FMS.

### 2.3. Outcome Measures

#### 2.3.1. Demographic Measures

All participants provided the following demographic and clinical information: gender, age, FM diagnosis onset, years of formal education and body mass index (BMI). The BMI index was calculated by dividing the weight by the square of the height.

#### 2.3.2. Cognitive Task

All cognitive tasks, except that of digits, were processed using the computer program E-Prime^®^ version 2.0 (Psychology Software Tools, Pittsburgh, PA, USA).

##### Memory Tasks: Working Memory

Digit Task

The Digit Span Task is a subtest belonging to the Wechsler Scale of Intelligence for Adults—WAIS [37], which measures the verbal component of working memory. It consists of two parts: digits in direct order and digits in reverse order. In both, the experimenter reads aloud a series of numbers (specifically, 7 pairs of sequences consisting of between 1 and 9 numbers that are incrementally increased) that the participant must repeat in the same order (direct condition) or in reverse order (reverse condition). The test ends when both attempts at a certain level fail.

Corsi Task

This task evaluates the spatial component of working memory. The task consists of two blocks: direct and inverse condition, respectively. Each trial begins with the appearance of a pattern of nine white squares on a gray background. These are colored red in a rapid sequence of two, three... up to nine squares in the direct condition, and eight in the reverse. After the sequence, the nine-square pattern appears again, and the participant must touch the squares that have changed color with the mouse in the same order (direct condition) in which this happened or in reverse order (reverse condition). Span (or capacity) memory was calculated based on the longest sequence that each participant recalled correctly, directly and inversely, in at least one of the two sequences. Reaction Times (RTs) of the sequences correctly reproduced in forward and reverse order were also calculated.

##### Attention Tasks

Go/No-Go Task

The Go/No-Go Task is a classical paradigm to investigate inhibition control [38]. The stimulus in this task was a rectangle presented in different corners of the screen. When the rectangle was presented in the upper left, upper right and lower right corners of the computer screen, these are known as Go trials, and when all the rectangles are presented in the lower left corner, these are No-Go trials. The participants were required to press the space bar for Go trials and not to press the space bar for No-Go trials. The error rate on the Go conditions, or errors of omission trials, and the percentage of errors in the No-Go conditions, or errors of commission, were analyzed. In addition, RTs obtained in Go trials by participants were taken into account by both groups.

Stroop Task with Negative Priming (NP)

This task was employed to evaluate inhibitory mechanisms and also interference effects in the NP condition [39]. Each trial started with the presentation of a fixation point (a cross) located in the center of the screen. Immediately afterwards, a word written in a determinate color appeared (for example, the word BLUE written in red ink). Participants had to press, as quickly as possible, the key that corresponded to the color of the ink in which the word was written (red), regardless of the word’s meaning. There were four possible colors (red, green, blue and yellow), and each was assigned to a key on the keyboard. Congruent trials were those in which the color of the word coincided with the color in which it was presented. Incongruent trials were those in which the color word did not coincide with the color in which it was displayed. Trials were also coded according to the congruency of the previous trial (N-1) in order to evaluate the NP effect for each trial. The measures of the RTs obtained by participants in congruent and incongruent trials were compared to calculate the Stroop effect. The negative priming effect was also calculated by comparing the measures of the RTs obtained by participants in control trials vs. incongruent trials.

### 2.4. Statistical Analyses 

Statistical analyses and graphics were performed using SPSS v19.0 (SPSS, Inc., Chicago, IL, USA) and GraphPad Prism v7.0 (GraphPad Software, La Jolla, CA, USA), respectively. All alpha levels were set at *p* < 0.05. As this was a pilot study, no power analysis was performed to predetermine sample size. 

First, a descriptive analysis was performed, and the normal distribution of variables was verified by the Kolmogorov–Smirnov test. Baseline demographics were compared between both groups using χ^2^ tests for categorical data and Student’s *t*-tests for continuous data. For the cognitive task, the mean scores (total and/or partial) were subjected to a repeated-measures analysis of variance (ANOVA). In addition, the Student’s *t*-test was employed to compare means between groups. 

Due to technical problems, some data were missing. The exact number of participants is indicated in each task. 

## 3. Results

### 3.1. Participant Characteristics

**Figure 1:**
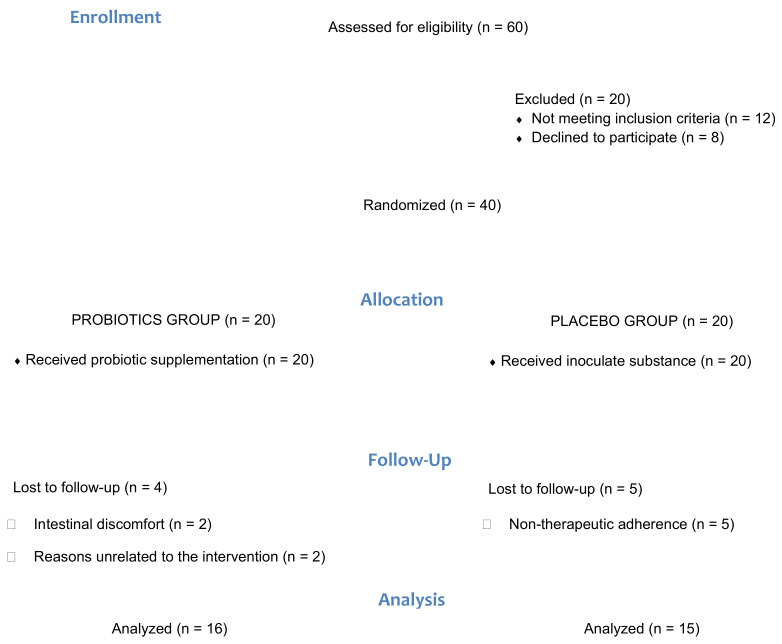
Flow diagram of the progress through the phases of the pilot parallel randomized trial of two groups.

A total of 31 patients diagnosed with FMS were allocated to the probiotic or placebo group (Figure 1). Sociodemographic variables are shown in Table 1, which describe the sample that participated in the study. No statistically significant differences in any of the variables between either group (*p* > 0.05) were observed.

### 3.2. Performance on Cognitive Task

#### 3.2.1. Memory Task

Digit Task

For each participant, the memory span (or capacity) score was calculated based on the longest sequence that was correctly remembered, forward and reverse (see Table 2), adding the corresponding score to all sequences answered correctly (two points were awarded when the two attempts of the sequence were reproduced correctly and one point when only one of them was remembered). These data were analyzed using an analysis of variance with one inter-subject manipulated factor, group (placebo, probiotic), and two within-subject manipulated factors, order (forward and reverse) and treatment (pre-, post-). No effect or interaction was statistically significant (*p* > 0.05).

Corsi Task

Span (or capacity) memory was calculated based on the longest sequence that each participant recalled correctly, directly and inversely, in at least one of the two sequences (Table 3). The average median RTs of the sequences correctly reproduced in forward and reverse order was also calculated (Table 4). These data were analyzed using analysis of variance with one factor manipulated between subjects, group (placebo, probiotic), and two factors manipulated within subjects, order (forward and reverse) and treatment (pre-, post-). No effect or interaction was statistically significant (*p* > 0.05).

#### 3.2.2. Attention Task

Go/No-Go Task

**Figure 2:**
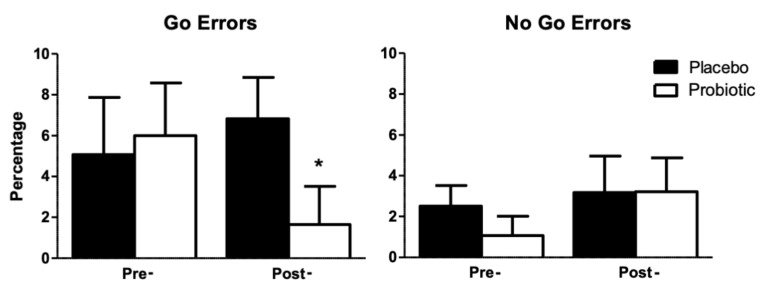
Percentage of omission errors (Go Errors) and of commission (No Go Errors) committed by participants in both groups depending on the treatment. Error bars represent the standard error of the mean. * marginal effect of the interaction group x treatment.

In this task, we analyzed the error rate for the Go conditions, or errors of omission trials, the percentage of errors in the No-Go conditions, or errors of commission, and the average of the medians of the RTs obtained in the Go trials (Table 5) by participants in both groups. These data were analyzed using analysis of variance with one factor manipulated between subjects, group (placebo, probiotic), and one factor manipulated within subjects, treatment (pre-, post-). The ANOVA of errors of omission showed a marginal effect of the interaction group x treatment (F1, 24 = 3.62; *p* = 0.069). Furthermore, a marginal effect of group (F1, 24 = 3.56; *p* = 0.071) was observed post-treatment in the Go condition (Figure 2). No other effect or interaction was statistically significant (*p* > 0.05).

Stroop Task

In this task, the measures of the median RTs obtained by participants in congruent and incongruent trials were compared to calculate Stroop effects. These data were analyzed using analysis of variance with two factors manipulated between subjects, group (placebo, probiotic) and condition (congruent, incongruent), and one factor manipulated within subjects, treatment (pre-, post-). The results only showed a significant effect of condition (*p* < 0.01); no other significant effects were observed (*p* > 0.05). The negative priming effect was also calculated by comparing the measures of the median RTs obtained by participants in control trials vs. incongruent trials. No effect or interaction was statistically significant (*p* > 0.05) (Table 6).

## 4. Discussion

The purpose of the present study was to continue exploring the beneficial effects of treatment with a multispecies probiotic in patients diagnosed with FMS. For this, a group of patients with a mean time of 8 and a half years since diagnosis and a mean age of 52 years were treated for 8 weeks with a multispecies probiotic or with a placebo substance and evaluated immediately for its effects on attention and memory.

To our knowledge, the only study evaluating the role of probiotics in cognition in FMS patients is our previous study, which showed a reduction in impulsivity after treatment [31]. In the current research, we found no significant differences in memory after treatment. Although no other studies have used probiotics to improve memory in FMS, a recent systematic review and meta-analysis of preclinical and clinical studies indicates that probiotics could be a useful strategy to improve dementia and cognitive decline [35] in both healthy [36] and elderly populations [40]. Similarly, a probiotic-treated Alzheimer’s experimental model demonstrated an improvement in learning [41] and memory [42]. In clinical studies of elderly people with mild cognitive impairment, an improvement in cognitive function (memory and attention) and an increase in brain-derived neurotrophic factor (BDNF) were reported after treatment with *Lactobacillus plantarum C29*-fermented soybean (DW2009) for 12 weeks [43]. Similar data were collected after the administration of *Bifidobacterium* A1 for 12 weeks in older adults with memory deficits, although the data are not conclusive and further research is required in this regard [44]. According to these studies, one possible explanation for the lack of positive results in our study could be the short length of treatment; studies demonstrating memory benefits were of significantly longer duration.

Regarding the attentional tasks, no differences in the Stroop effect or the negative priming effect (Stroop Task with Negative Priming) were observed among the participants after the treatment, implying that the probiotic treatment used did not affect the inhibitory mechanisms of attention. However, patients with FMS treated with the probiotic showed a tendency towards reduced errors of omission (Go trials) during the Go/No-Go Task and the group that received the placebo presented a number of errors that was slightly higher than those registered in the pre-treatment phase. This type of error occurs when there is an absence of response to a relevant stimulus, and it is assumed that it reflects symptoms of inattention [45]. Therefore, FMS patients treated and not treated with the probiotic showed similar levels of inhibitory motor control and similar ability to inhibit information irrelevant to the task objective, but they differed in their ability to maintain attention for an extended period with the objective of responding to specific stimuli. This difference could be attributed to the effect that probiotics produced in these patients, which improved their ability to maintain attention, as evidenced by the results obtained in the Go/No-Go Task in the post-treatment phase.

Despite studies finding that the effects of probiotics on attention are reduced, similar results have been observed in other populations. In this regard, after 8 weeks of treatment with *Lactobacillus plantarum* 299v, patients with major depression showed an improvement in attention and work speed on the attention and perceptivity test, but no significant effects on the Stroop test [46]. Similarly, *Lactobacillus plantarum* DR7 treatment for 12 weeks improved basic attention and memory in healthy adults, as measured by the computerized CogState Brief Battery [47].

A recent systematic review and meta-analysis showed a positive effect of probiotics on cognition in both humans and animals [48]. Human studies showed an improvement in attention and memory in patients with Alzheimer’s, in the healthy elderly individuals or those with depression. The only FMS study included in this analysis was the first part of our current research [31]. Most included studies used *Lactobacillus* and *Bifidobacterium* probiotic strains, but it is worth noting that the meta-analysis found that using just one probiotic was more effective than using a combination. In the same manner, the 12-week treatment was more effective than the 8-week treatment, implying that our findings on FM cognition could be significant after additional weeks of treatment.

The putative mechanisms of action of probiotics in cognitive function, as suggested by Lv and collaborators [48], are related to neuroinflammation. In this regard, the decline in cognitive function associated with aging is related to changes in brain immunoregulation, including decreases in IL-4 [49]. Several studies suggest a decrease in the diversity of microbiota with cognition and inflammatory markers [50], in which changes in the intestinal metagenome appear to be associated with cognitive function and brain iron deposition [51]. In this context, factors associated with aging, such as oxidative stress and inflammation, are related to the intestinal microbiota [52], which influences the different sequences of cognitive impairment [53], and probiotic treatment could reverse this cognitive impairment via cytokine systems.

Interestingly, elevations of proinflammatory chemokines/cytokines could negatively impact symptoms of FMS. Proinflammatory cytokines have been shown to have an important modulatory role in pain transmission and perception. It is not surprising that high levels of them have been found, specifically of interleukins 1, 2, 6 and 8, in patients with FMS [54]. Therefore, probiotic administration could be an effective approach to treat cognitive deficits in FMS, as can be seen in our results. In other words, a multispecies probiotic treatment can improve some cognitive functions in FMS patients, such as impulse control, sustained attention and the ability to maintain attentional control in a context of change. The clinical relevance of microbiota modulation in FMS patients should be considered as an adjuvant treatment.

However, these results must be taken with caution, given that this study had several limitations. First of all, we had a limited number of subjects, since this was a pilot randomized controlled trial. Secondly, the nutritional habits of the participants should have been registered because they could influence or interfere with the results—for example, the effect of the consumption of other fermented foods. Finally, measuring the gut microbiota would have given us more information about probiotic modulation. In this manner, future studies should be designed with a large sample size while keeping these limitations in mind. 

## 5. Conclusions

Treatment of FMS patients with a multispecies probiotic for 8 weeks resulted in a tendency towards fewer errors in attention to relevant stimuli, particularly in a task that required inhibitory control at the motor level. However, this treatment had no effect on memory, specifically on working memory. These findings, along with those of our previous research on impulsivity, point to the importance of using probiotics as a therapeutic option in FMS. Nonetheless, more research is needed given the potential role of probiotics in FMS, especially since dysbiosis has been reported in FMS patients. In future studies, authors should consider exploring the effect of a specific probiotic strain on the treatment of cognitive impairment.

## Figures and Tables

**Table 1:** Sociodemographic characteristics of study population.

	PROBIOTIC (*n* = 16)	PLACEBO (*n* = 15)
Gender (%)		
Men	6.25	13.33
Women	93.75	86.67
Age	55.00 ± 8.37	50.27 ± 7.86
Years of diagnosis	8.56 ± 5.90	8.47 ± 5.80
Formal education (years)	12.75 ± 0.95	12.27 ± 1.29
BMI (kg/m^2^)	29.40 ± 1.64	30.23 ± 1.63

**Table 2:** Span of memory expressed by mean and standard error.

	PLACEBO (*n* = 15)		PROBIOTIC (*n* = 16)	
	PRE-	POST-	PRE-	POST-
Forward	8.42 (0.48)	8.78 (0.48)	8.06 (0.54)	8.31 (0.59)
Reverse	5.35 (0.26)	5.50 (0.47)	5.56 (0.60)	5.62 (0.56)

**Table 3:** Span of memory expressed by mean and standard error.

	PLACEBO (*n* = 12)		PROBIOTIC (*n* = 13)	
	PRE-	POST-	PRE-	POST-
Forward	4.92 (337)	5.08 (37)	5.00 (311)	5.39 (288)
Reverse	4.50 (324)	4.83 (299)	5.08 (356)	4.92 (356)

**Table 4:** The average median of Reaction Times (RTs) expressed by mean and standard error.

	PLACEBO (*n* = 12)		PROBIOTIC (*n* = 13)	
	PRE-	POST-	PRE-	POST-
Forward	4602 (378)	4600 (474)	3823 (362)	3839 (454)
Reverse	3998 (316)	4520 (417)	3911 (303)	3615 (399)

**Table 5:** The average median of RTs expressed by mean and standard error.

	PLACEBO (*n* = 12)		PROBIOTIC (*n* = 14)	
	PRE-	POST-	PRE-	POST-
Go trials	400 (47.3)	378 (35.7)	344 (43.8)	365 (33.1)

**Table 6:** The average median of RTs and errors expressed by mean and standard error.

	PLACEBO (*n* = 11)		PROBIOTIC (*n* = 12)	
	PRE-	POST-	PRE-	POST-
*RTs*				
Congruent	1013 (60)	998 (59)	987 (58)	979 (57)
Incongruent	1094 (69)	1044 (61)	1075 (66)	1065 (58)
Control	1076 (68)	1036 (57)	1061 (66)	1045 (55)
Ignored	1051 (72)	1023 (69)	1052 (69)	1050 (66)
*Errors*				
Congruent	0.6 (0.4)	0.5 (0.2)	0.5 (0.4)	0.7 (0.2)
Incongruent	3.1 (2.8)	2.3 (0.9)	4.7 (2.7)	1.6 (0.8)
Control	2.5 (1.6)	2.5 (0.9)	4.8 (2.5)	0.7 (0.9)
Ignored	3.4 (2.5)	1.4 (0.5)	4.4 (2.4)	0.7 (0.5)

## Data Availability

The data presented in this study are available on request from the corresponding author. The data are not publicly available due to ethical considerations.

## References

[1] Wolfe F., Smythe H.A., Yunus M.B., Bennett R.M., Bombardier C., Goldenberg D.L., Tugwell P., Campbell S.M., Abeles M., Clark P. (1990). The American College of Rheumatology 1990 Criteria for the Classification of Fibromyalgia. Report of the Multicenter Criteria Committee. Arthritis Rheum..

[2] Glass J.M. (2008). Fibromyalgia and cognition. J. Clin. Psychiatry.

[3] Glass J.M. (2009). Review of Cognitive Dysfunction in Fibromyalgia: A Convergence on Working Memory and Attentional Control Impairments. Rheum. Dis. Clin. N. Am..

[4] Verdejo-García A., López-Torrecillas F., Calandre E.P., Delgado-Rodríguez A., Bechara A. (2009). Executive function and decision-making in women with fibromyalgia. Arch. Clin. Neuropsychol..

[5] Park D.C., Glass J.M., Minear M., Crofford L.J. (2001). Cognitive Function in Fibromyalgia Patients. Arthritis Rheum..

[6] Leavitt F., Katz R.S. (2009). Normalizing memory recall in fibromyalgia with rehearsal: A distraction-counteracting effect. Arthritis Care Res..

[7] Glass J.M., Williams D.A., Fernandez-Sanchez M.L., Kairys A., Barjola P., Heitzeg M.M., Clauw D.J., Schmidt-Wilcke T. (2011). Executive function in chronic pain patients and healthy controls: Different cortical activation during response inhibition in fibromyalgia. J. Pain.

[8] Minerbi A., Gonzalez E., Brereton N.J.B., Anjarkouchian A., Dewar K., Fitzcharles M.A., Chevalier S., Shir Y. (2019). Altered microbiome composition in individuals with fibromyalgia. Pain.

[9] Freidin M.B., Stalteri M.A., Wells P.M., Lachance G., Baleanu A.-F., Bowyer R.C.E., Kurilshikov A., Zhernakova A., Steves C.J., Williams F.M.K. An association between chronic widespread pain and the gut microbiome. Rheumatology.

[10] Moloney R.D., Johnson A.C., O’Mahony S.M., Dinan T.G., Greenwood-Van Meerveld B., Cryan J.F. (2016). Stress and the Microbiota-Gut-Brain Axis in Visceral PaRelevance to Irritable Bowel Syndrome. CNS Neurosci. Ther..

[11] Logan A.C., Katzman M. (2005). Major depressive disorder: Probiotics may be an adjuvant therapy. Med. Hypotheses.

[12] Butt H., Dunstan R., McGregor N., Roberts T. Bacterial colonosis in patients with persistent fatigue. Proceedings of the AHMF International Clinical and Scientific Conference.

[13] Mayer E.A., Tillisch K., Gupta A., Mayer E.E.A., Rhee S., Pothoulakis C., Mayer E.E.A., Cryan J., Dinan T., Mayer E.E.A. (2015). Gut/brain axis and the microbiota. J. Clin. Investig..

[14] Bercik P., Collins S.M. (2014). The effects of inflammation, infection and antibiotics on the microbiota-gut-brain axis. Adv. Exp. Med. Biol..

[15] De Palma G., Collins S.M., Bercik P., Verdu E.F. (2014). The microbiota-gut-brain axis in gastrointestinal disorders: Stressed bugs, stressed brain or both?. J. Physiol..

[16] Al-Nedawi K., Mian M.F., Hossain N., Karimi K., Mao Y.-K., Forsythe P., Min K.K., Stanisz A.M., Kunze W.A., Bienenstock J. (2015). Gut commensal microvesicles reproduce parent bacterial signals to host immune and enteric nervous systems. FASEB J..

[17] Chichlowski M., Rudolph C. (2015). Visceral pain and gastrointestinal microbiome. J. Neurogastroenterol. Motil..

[18] Dinan T.G., Stanton C., Cryan J.F. (2013). Psychobiotics: A novel class of psychotropic. Biol. Psychiatry.

[19] WHO (2001). Report of the Joint FAO/WHO Expert Consultation on Evaluation of Health and Nutritional Properties of Probiotics in Food Including Powder Milk with Live Lactic Acid Bacteria, Córdoba, Argentina, 1–4 October 2001.

[20] Hardy H., Harris J., Lyon E., Beal J., Foey A.D. (2013). Probiotics, prebiotics and immunomodulation of gut mucosal defences: Homeostasis and immunopathology. Nutrients.

[21] Wichmann A., Allahyar A., Greiner T.U., Plovier H., Lundén G.Ö., Larsson T., Drucker D.J., Delzenne N.M., Cani P.D., Bäckhed F. (2013). Microbial Modulation of Energy Availability in the Colon Regulates Intestinal Transit. Cell Host Microbe.

[22] Minerbi A., Fitzcharles M.A. (2020). Gut microbiome: Pertinence in fibromyalgia. Clin. Exp. Rheumatol..

[23] Rodrigo L., Blanco I., Bobes J., De Serres F.J. (2014). Effect of one year of a gluten-free diet on the clinical evolution of irritable bowel syndrome plus fibromyalgia in patients with associated lymphocytic enteritis: A case-control study. Arthritis Res. Ther..

[24] Pusceddu M.M., Murray K., Gareau M.G. (2018). Targeting the Microbiota, From Irritable Bowel Syndrome to Mood Disorders: Focus on Probiotics and Prebiotics. Curr. Pathobiol. Rep..

[25] Penfold S., Denis E.S., Mazhar M.N. (2016). The association between borderline personality disorder, fibromyalgia and chronic fatigue syndrome: Systematic review. BJPsych Open.

[26] Roman P., Carrillo-Trabalón F., Sánchez-Labraca N., Cañadas F., Estévez A.F., Cardona D. (2018). Are probiotic treatments useful on fibromyalgia syndrome or chronic fatigue syndrome patients? A systematic review. Benef. Microbes.

[27] Nelson J., Sjöblom H., Gjertsson I., Ulven S.M., Lindqvist H.M., Bärebring L. (2020). Do Interventions with Diet or Dietary Supplements Reduce the Disease Activity Score in Rheumatoid Arthritis? A Systematic Review of Randomized Controlled Trials. Nutrients.

[28] Erdrich S., Hawrelak J.A., Myers S.P., Harnett J.E. (2020). Determining the association between fibromyalgia, the gut microbiome and its biomarkers: A systematic review. BMC Musculoskelet. Disord..

[29] Pagliai G., Giangrandi I., Dinu M., Sofi F., Colombini B. (2020). Nutritional interventions in the management of fibromyalgia syndrome. Nutrients.

[30] Roman P., Estévez Á.F., Sánchez-Labraca N., Cañadas F., Miras A., Cardona Mena D. (2017). Probióticos en fibromialgia: Diseño de un estudio piloto doble ciego y randomizado. Nutr. Hosp..

[31] Roman P., Estévez A.F., Miras A., Sánchez-Labraca N., Cañadas F., Vivas A.B., Cardona D. (2018). A Pilot Randomized Controlled Trial to Explore Cognitive and Emotional Effects of Probiotics in Fibromyalgia. Sci. Rep..

[32] Wolfe F., Clauw D.J., Fitzcharles M.-A., Goldenberg D.L., Katz R.S., Mease P., Russell A.S., Russell I.J., Winfield J.B., Yunus M.B. (2010). The American College of Rheumatology preliminary diagnostic criteria for fibromyalgia and measurement of symptom severity. Arthritis Care Res..

[33] Kato-Kataoka A., Nishida K., Takada M., Suda K., Kawai M., Shimizu K., Kushiro A., Hoshi R., Watanabe O., Igarashi T. (2016). Fermented milk containing *Lactobacillus casei* strain Shirota prevents the onset of physical symptoms in medical students under academic examination stress. Benef. Microbes.

[34] Kelly J.R., Allen A.P., Temko A., Hutch W., Kennedy P.J., Farid N., Murphy E., Boylan G., Bienenstock J., Cryan J.F. (2017). Lost in translation? The potential psychobiotic Lactobacillus rhamnosus (JB-1) fails to modulate stress or cognitive performance in healthy male subjects. Brain. Behav. Immun..

[35] Ruiz-Gonzalez C., Roman P., Rueda-Ruzafa L., Rodriguez-Arrastia M., Cardona D. (2020). Effects of probiotics supplementation on dementia and cognitive impairment: A systematic review and meta-analysis of preclinical and clinical studies. Prog. Neuro-Psychopharmacol. Biol. Psychiatry.

[36] Benton D., Williams C., Brown A. (2007). Impact of consuming a milk drink containing a probiotic on mood and cognition. Eur. J. Clin. Nutr..

[37] Ibor J. (2005). Escala de Inteligencia de Wechsler para Adultos III. Schizophr. Res..

[38] Casey B.J., Trainor R.J., Orendi J.L., Schubert A.B., Nystrom L.E., Giedd J.N., Castellanos F.X., Haxby J.V., Noll D.C., Cohen J.D. (1997). A developmental functional MRI study of prefrontal activation during performance of a Go-No-Go task. J. Cogn. Neurosci..

[39] Mayas J., Fuentes L.J., Ballesteros S. (2012). Stroop interference and negative priming (NP) suppression in normal aging. Arch. Gerontol. Geriatr..

[40] Inoue T., Kobayashi Y., Mori N., Sakagawa M., Xiao J.Z., Moritani T., Sakane N., Nagai N. (2018). Effect of combined bifidobacteria supplementation and resistance training on cognitive function, body composition and bowel habits of healthy elderly subjects. Benef. Microbes.

[41] Rezaeiasl Z., Salami M., Sepehri G. (2019). The effects of probiotic Lactobacillus and Bifidobacterium strains on memory and learning behavior, long-term potentiation (LTP), and some biochemical parameters in β-amyloid-induced rat’s model of Alzheimer’s disease. Prev. Nutr. Food Sci..

[42] Rezaei Asl Z., Sepehri G., Salami M. (2019). Probiotic treatment improves the impaired spatial cognitive performance and restores synaptic plasticity in an animal model of Alzheimer’s disease. Behav. Brain Res..

[43] Hwang Y.H., Park S., Paik J.W., Chae S.W., Kim D.H., Jeong D.G., Ha E., Kim M., Hong G., Park S.H. (2019). Efficacy and safety of lactobacillus plantarum C29-fermented soybean (DW2009) in individuals with mild cognitive impairment: A 12-week, multi-center, randomized, double-blind, placebo-controlled clinical trial. Nutrients.

[44] Kobayashi Y., Kuhara T., Oki M., Xiao J.Z. (2019). Effects of bifidobacterium breve a1 on the cognitive function of older adults with memory complaints: A randomised, double-blind, placebo-controlled trial. Benef. Microbes.

[45] Barkley R.A. (1997). Behavioral inhibition, sustained attention, and executive functions: Constructing a unifying theory of ADHD. Psychol. Bull..

[46] Rudzki L., Ostrowska L., Pawlak D., Małus A., Pawlak K., Waszkiewicz N., Szulc A. (2019). Probiotic Lactobacillus Plantarum 299v decreases kynurenine concentration and improves cognitive functions in patients with major depression: A double-blind, randomized, placebo controlled study. Psychoneuroendocrinology.

[47] Chong H.X., Yusoff N.A.A., Hor Y.Y., Lew L.C., Jaafar M.H., Choi S.B., Yusoff M.S.B., Wahid N., Abdullah M.F.I.L., Zakaria N. (2019). Lactobacillus plantarum DR7 alleviates stress and anxiety in adults: A randomised, double-blind, placebo-controlled study. Benef. Microbes.

[48] Lv T., Ye M., Luo F., Hu B., Wang A., Chen J., Yan J., He Z., Chen F., Qian C. (2021). Probiotics treatment improves cognitive impairment in patients and animals: A systematic review and meta-analysis. Neurosci. Biobehav. Rev..

[49] Frank M.G., Fonken L.K., Watkins L.R., Maier S.F., Lowry C.A. (2019). Could Probiotics Be Used to Mitigate Neuroinflammation?. ACS Chem. Neurosci..

[50] Claesson M.J., Cusack S., O’Sullivan O., Greene-Diniz R., De Weerd H., Flannery E., Marchesi J.R., Falush D., Dinan T., Fitzgerald G. (2011). Composition, variability, and temporal stability of the intestinal microbiota of the elderly. Proc. Natl. Acad. Sci. USA.

[51] Blasco G., Moreno-Navarrete J.M., Rivero M., Pérez-Brocal V., Garre-Olmo J., Puig J., Daunis-i-Estadella P., Biarnés C., Gich J., Fernández-Aranda F. (2017). The gut metagenome changes in parallel to waist circumference, brain iron deposition, and cognitive function. J. Clin. Endocrinol. Metab..

[52] Heyck M., Ibarra A. (2019). Microbiota and memory: A symbiotic therapy to counter cognitive decline?. Brain Circ..

[53] Brüssow H. (2013). Microbiota and healthy ageing: Observational and nutritional intervention studies. Microb. Biotechnol..

[54] Theoharides T.C., Tsilioni I., Bawazeer M. (2019). Mast Cells, Neuroinflammation and Pain in Fibromyalgia Syndrome. Front. Cell. Neurosci..

